# Hope and expectancies for future events in depression

**DOI:** 10.3389/fpsyg.2013.00470

**Published:** 2013-07-24

**Authors:** Jens C. Thimm, Arne Holte, Tim Brennen, Catharina E. A. Wang

**Affiliations:** ^1^Department of Psychology, University of TromsøTromsø, Norway; ^2^Helgeland Hospital TrustMo i Rana, Norway; ^3^Division of Mental Health, Norwegian Institute of Public HealthOslo, Norway; ^4^Department of Psychology, University of OsloOslo, Norway

**Keywords:** depression, dysfunctional attitudes, prospective cognitions, hope, unrealistic optimism

## Abstract

The present study investigated prospective cognition with the Hope scale (Snyder et al., 1991) and the Unrealistic Optimism Scale (Weinstein, 1980) in clinically depressed (CD; *n* = 61), previously depressed (PD; *n* = 42), and never depressed controls (ND; *n* = 46). In line with previous research, significant negative correlations between hope and symptoms of depression were found. Previously depressed reported lower levels of hope than NDs, but were more hopeful than CDs. In addition, relationships between depressive symptoms, dysfunctional attitudes, and expectations for the future were examined. As hypothesized, the CDs estimated their probability of experiencing positive events in the future as lower and their probability of experiencing negative events as higher than the two other groups. The PDs differed not from the NDs in their probability estimates. Implications of the findings are discussed.

Conceptualizations of depression often include an emphasis on the role of negative future-oriented cognitions. For example, hopelessness about the future is central in cognitive accounts of depression (e.g., Beck et al., 1979; Abramson et al., 1989). Beck (1976) has suggested that depression is characterized by a negative cognitive triad consisting of negative views of the self, the personal world, and the future. Also, Beck suggested that depression prone individuals are characterized by negative self-schemas, containing dysfunctional attitudes, frequently assessed with the Dysfunctional Attitude Scale (DAS; Weissman and Beck, 1978). The DAS includes core beliefs such as that one's happiness depends on being perfect or on other people's approval (de Graaf et al., 2009). Abramson et al. (1989) reformulated the revised learned helplessness theory in terms of hopelessness and proposed that depression-prone individuals attribute negative life events to internal, stable, and global causes. Alloy and Ahrens (1987) demonstrated that a depressogenic attribution style is associated with pessimism regarding the future.

Several studies have examined prospective cognition in depression and found reduced positive expectations in this group. For example, using a verbal fluency paradigm, MacLeod et al. (1997b) found that depressed individuals generated significantly fewer positive events than anxious and controls. There were no group differences in a verbal fluency control task, making it unlikely that the cognitive deficits often found in depressed patients can explain this result. Recently, this finding has been replicated in mild to moderate depressed outpatients (Bjärehed et al., 2010). The effect is probably due to difficulties in assessing mental representations of positive future experiences (MacLeod and Salaminiou, 2001). Furthermore, mental images of positive prospective events of depressed individuals are characterized by lower vividness (Morina et al., 2011).

Hope and optimism are overlapping, but distinguishable future-oriented constructs that have received considerable attention in the psychological literature (Alarcon et al., 2013). Snyder (1995) ties hopeful thinking expressly to goals and regards goal-directed thoughts as the basic building blocks for human learning and coping. According to Snyder (1995), goal-directed cognitions include the cognitive willpower or energy to get moving toward one's goal (the agency component) and the perceived ability to generate routes to get to a goal (the pathway component). He suggests that both the agentic and pathway goal-related thoughts are necessary to yield hope and that blockage to either pathway or agency thought may have an impact on coping. Both the agency and pathways components have been found to be negatively correlated with depression (e.g., Chang, 2003). Furthermore, hope has been found to moderate the effects of, for example, rumination and negative life events on depressive symptoms (Geiger and Kwon, 2010; Visser et al., 2013). However, only the agency component had a unique effect on later depression in a study conducted by Arnau et al. (2007).

Optimism has been shown to be beneficial for mental and physical health (Peterson, 2000). Unrealistic optimism refers to an overestimation of the likelihood of experiencing positive events in the future and an underestimation of the probability of experiencing negative events as to compared to others (Weinstein and Klein, 1996; Sharot, 2011). A number of studies have supported the presence of unrealistic optimism in normal, healthy individuals (e.g., Weinstein, 1980, 1987; Harris and Middleton, 1994; Campbell et al., 2007). For example, Weinstein (1980) found college students to rate their own chances, as opposed to classmates, to be above average for positive events and below average for negative events. A possible explanation of the mechanism responsible for the unrealistic optimism phenomena may be the illusion of control. This suggestion was actually supported by Harris and Middleton (1994) who found the unrealistic optimism to be associated with the perceived controllability of the negative event. Overly optimistic beliefs have been suggested to be important for mental and physical health (Taylor and Brown, 1988; Taylor et al., 2000). In contrast to hope, optimism is considered a personality trait. According to the five-factor model (Costa and McCrae, 1992), optimism is a trait of extraversion. Studies have confirmed the strong relationship with extraversion (Sharpe et al., 2011) and shown that optimism has higher correlations with personality traits than hope (Alarcon et al., 2013).

Research on subjective probability judgments has demonstrated that people with anxiety and depression differ from normal, healthy individuals. They tend to judge negative future events as more likely to happen to them than to others (Butler and Mathews, 1983; MacLeod and Cropley, 1995; MacLeod et al., 1997b). Also, mood-disturbed individuals have been found to judge positive future events as less likely to happen to them than to others (Pyszczynski et al., 1987; MacLeod and Cropley, 1995; MacLeod et al., 1997b). Furthermore, the positivity bias commonly found in healthy individuals is attenuated in mood-disturbed individuals (Moore and Fresco, 2012). Recently, interventions have been developed that aim to enhance positive expectations in depressed individuals (Vilhauer et al., 2012).

However, little is known about hope and optimism in individuals who have recovered from depression. Therefore, the purpose of the present study is to explore and compare hope and unrealistic optimism in never depressed, previously depressed, and clinically depressed individuals. Due to the previous experience of losing control (i.e., the depressive breakdown) it is expected that the unrealistic optimism about future events observed in normal, healthy individuals would not be present to the same degree in formerly depressed individuals. Also, we wanted to explore the relationship between dysfunctional attitudes and hope and probability estimates of positive and negative future events, respectively.

## Methods

### Participants

The current investigation is part of a research project on cognitive vulnerability for depression (Wang, 2006). Participants were 149 subjects, 122 women and 27 mens, aged 18–54 (*M* = 28.6, *SD* = 10). The recruitment and diagnosis procedures have been described in detail in Wang et al. (2005). In brief, participants were recruited among undergraduate students and patients consulting their general practitioner and screened with the Beck Depression Inventory (BDI; Beck et al., 1979) and the Previous Depression Questionnaire (PDQ; Wang, 1996). Out of about 340 students and 180 patients who returned the questionnaires and were willing to take part in the study, 184 subjects (84 patients and 100 students) were invited to participate. These individuals had a BDI score above 16 (clinically depressed) or had a lower score, but met the requirements for previous depression on the PDQ. In addition, a sample was randomly drawn from those who had a BDI score in the normal range (between 0 and 9) and did not report previous depression on the PDQ. Participants were diagnosed using the mood disorder and psychotic symptoms sections of the SCID I (First et al., 1997). Individuals were excluded if the diagnostic criteria for a current or previous depressive episode were not met, the depressive episode was more than five years ago, or psychotic or hypomanic symptoms were present (*N* = 30). Additional five individuals dropped out before study completion. The final sample consisted of 61 clinically depressed (CD; 52 women and 9 men; *M* = 30.8 years, *SD* = 10), 42 previously depressed (PD; 35 women and 7 men; *M* = 27.0 years, *SD* = 8) and 46 never depressed controls (ND; 35 women and 11 men; *M* = 26.9 years, *SD* = 9).

### Measures

#### Dysphoric symptoms

Dysphoric symptoms were measured with the BDI (Beck et al., 1979), which is a widely used 21-item self-report symptom scale that assesses a variety of affective, behavioral, cognitive, and somatic symptoms indicating dysphoric states or clinical depression. For each item, there are four alternative statements, which reflect increasing levels of severity. Possible scores range from 0 to 63. Psychometric properties of the BDI have been provided by Beck et al. (1988).

#### Dysfunctional attitudes

Dysfunctional attitudes were assessed with the Dysfunctional Attitude Scale (Form A) (DAS; Weissman and Beck, 1978). The DAS is a 40-item self-report inventory designed to measure the presence of dysfunctional attitudes. The content of these statements concern need for approval, perfectionism, and rigid ideas about the world. Items are rated on a 7-point scale ranging from “totally agree” to “totally disagree.”

#### Hope scale

The Hope Scale (Snyder et al., 1991) is a 12-item self-report scale designed to measure dispositional hope. The scale exists of four items tapping agency for goals, four items measuring pathways for goals, and four distracters. Each of the twelve items is rated according to a Likert-type scale that ranges from 1 (definitely false) to 4 (definitely true). The total score is the sum of the agency and pathways items scores. Snyder et al. (1991) report that the Hope Scale has adequate internal and test-retest reliability. In addition, support for a two-factor model of the scale with the agency and pathways components has been found in confirmatory factor analyses (Babyak et al., 1993).

#### Unrealistic optimism scale

The Unrealistic Optimism Scale (UOS; Weinstein, 1980) is an 84-item self-report scale designed to measure unrealistic optimism for future events. The UOS consists of 18 positive and 24 negative events. The 42 positive and negative items are rated twice. First, with the instruction: “Please, try to estimate in percent (between 0 and 100%) how probable it is that the following events will happen to you?” And second with the instruction: “Please, try to estimate in percent (between 0 and 100%) how probable it is that the following events will happen to a person of the same sex as you?”. Examples of positive and negative UOS items are “Not ill all winter” and “Tripping and breaking bone”.

## Results

As reported previously (Wang et al., 2005), there were significant differences between the CDs, PDs, and NDs with respect to dysphoric symptoms and dysfunctional attitudes.

Means and standard deviations of the Hope and UOS scales in the three groups are shown in Table 1. To determine whether the three groups of participants differed with respect to hope and estimated probability for future events, separate one-way analyses of variance (ANOVA) were performed. Levene's test for equality of variances was significant for the hope total score (*p* < 0.001), hope agency (*p* < 0.01), hope pathways (*p* < 0.01), and estimation of the probability that others will experience positive events (*p* < 0.05). Following the recommendation by Field (2009), the Welch *F*-ratio is reported for these variables. *Post-hoc* comparisons were conducted using the Games-Howell test.

**Table 1:** **Means and standard deviations of Hope and UOS scales in clinically depressed, previously depressed and never depressed**.

	**Never depressed**		**Previously depressed**		**Clinically depressed**	
	***M***	***SD***	***M***	***SD***	***M***	***SD***
Hope_total_	3.14	0.32	2.85	0.38	2.43	0.52
Hope_agency_	3.12	0.35	2.84	0.41	2.41	0.58
Hope_pathways_	3.17	0.38	2.86	0.49	2.45	0.58
UOS_own probability of pos. events_	42.19	12.80	39.44	10.49	33.87	13.07
UOS_own probability of neg. events_	21.56	9.37	22.75	10.71	28.70	12.68
UOS_others probability of pos. events_	36.88	11.93	38.16	10.46	43.58	16.58
UOS_others probability of neg. events_	24.73	11.08	23.08	13.03	24.81	14.50

^*^p < 0.05; ^**^p < 0.01.

UOS_own probability of pos. events_, estimate of own probability of experiencing positive events in the future;

UOS_own probability of neg. events_, estimate of own probability of experiencing negative events in the future;

UOS_others probability of pos. events_, estimate of others probability of experiencing positive events in the future;

UOS_others probability of neg. events_, estimate of others probability of experiencing negative events in the future.

These analyses yielded a significant effect for group on the total hope score, *F*_(2, 94.98)_ = 38.90, *p* < 0.001, on the hope agency, *F*_(2, 95.31)_ = 31.24, *p* < 0.001, and on the hope pathways scales, *F*_(2, 93.67)_ = 29.94, *p* < 0.001. *Post-hoc* tests indicate that all three groups differed significantly from each other on all three hope measures (all *p*s < 0.01). The clinically depressed reported less hope than the previously depressed who again had lower scores on the hope scales than the never depressed.

Clinically depressed, previously depressed, and never depressed controls differed significantly in their estimates of their own and others probability of experiencing positive events in the future, *F*_(2, 146)_ = 6.39, *p* < 0.01 and *F*_(2, 96.96)_ = 3.17, *p* < 0.05, respectively. The three groups also differed significantly in their estimates of their own probability of experiencing negative events in the future, *F*_(2, 146)_ = 6.35, *p* < 0.01, but not the probability of others, *F*_(2, 145)_ = 0.25, *p* = 0.779.

The clinically depressed estimated their probability of experiencing positive events in the future as lower and their probability of experiencing negative events as higher than the previously depressed and never depressed. *Post-hoc* comparisons showed significant differences between the CDs and PDs (*p* < 0.05) and NDs (*p* < 0.01), but not between the last two groups. With respect to estimates of the probability that others will experience positive events in the future, there was a significant difference between the CDs and NDs (*p* < 0.05).

To further investigate the relationships of depression with dysfunctional attitudes, hope, probability estimates of future events, a multiple regression analysis was conducted with the BDI as the dependent variable. The results indicated that the predictors explained 55.3% of the variance [*F*_(7, 139)_ = 24.55, *p* < 0.001]. Individual significant predictors were dysfunctional attitudes (β = 0.27, *p* < 0.001), hope agency (β = −0.24, *p* < 0.01), and estimates of others probability of experiencing positive events in the future (β = 0.16, *p* < 0.05).

T-tests within each group showed that participants in all three groups estimated their own probability to experience positive life events as higher than their probability to experience negative life events [ND: *t*_(45)_ = 9.30, *p* < 0.001; PD: *t*_(41)_ = 6.92, *p* < 0.001; CD: *t*_(60)_ = 2.27, *p* < 0.05]. Further, participants in all three groups estimated another person's probability to experience positive events as higher than their probability to experience negative events [ND: *t*_(45)_ = 5.58, *p* < 0.001; PD: *t*_(41)_ = 7.29, *p* < 0.001; CD: *t*_(59)_ = 8.04, *p* < 0.001]. Finally, NDs estimated that their own probability for experiencing positive events in the future is higher than for others [*t*_(45)_ = 2.77, *p* < 0.01], while CDs estimated that another person's probability is higher than their own [*t*_(60)_ = −4.05, *p* < 0.001]. No significant difference was obtained for the PDs [*t*_(41)_ = 0.64, *p* = 0.527]. With respect to negative events, NDs estimated their own probability as less than for another person [*t*_(45)_ = −2.77, *p* < 0.01], while CDs estimated another person's probability as less than their own probability [*t*_(59)_ = 2.52, *p* < 0.05]. Again, no significant difference was obtained for the PDs [*t*_(41)_ = −0.22, *p* = 0.825].

Correlations between the BDI, DAS, Hope scales, and UOS scales in the total sample are displayed in Table 2. Dysphoric symptoms and dysfunctional attitudes were highly and negatively correlated with the three hope scales. The BDI and DAS scores were negatively related to estimates of one's own probability of experiencing positive events, but positively to estimates of one's own probability of experiencing negative events and others probability of experiencing positive events. There were no significant correlations between dysphoric symptoms and dysfunctional attitudes and estimates of others probability of experiencing negative events in the future. Correlations between the three hope variables and four UOS scales are also shown in Table 2. The hope scales showed a reverse pattern of relations with probability estimates of future events as dysphoric symptoms and dysfunctional attitudes. However, there was a small, but significant positive correlation between hope pathways and estimates of others probability of experiencing negative events. To check whether depressive symptoms account for the high number of significant associations, correlational analyses were rerun controlling for BDI scores. Results showed generally reduced correlation coefficients. Associations between the DAS and UOS scales were no longer significant at the 5% level. Except for a positive correlation between hope pathways and estimates of others probability of negative events, the Hope Scales were only significantly and positively related to estimates of own probability of positive events in the future.

**Table 2:** **Correlations between the BDI, DAS, UOS, and Hope Scale in the total sample**.

	**1**	**2**	**3**	**4**	**5**	**6**	**7**	**8**	**9**
1. BDI	–								
2. DAS	0.60^**^	–							
3. Hope_total_	−0.65^**^	−0.60^**^	–						
4. Hope_agency_	−0.59^**^	−0.48^**^	0.91^**^	–					
5. Hope_ pathways_	−0.60^**^	−0.62^**^	0.92^**^	0.67^**^	–				
6. UOS_own probability of pos. events_	−0.34^**^	−0.20^*^	0.46^**^	0.46^**^	0.39^**^	–			
7. UOS_own probability of neg. events_	0.37^**^	0.31^**^	−0.26^**^	−0.26^**^	−0.22^**^	−0.05	–		
8. UOS_others probability of pos. events_	0.32^**^	0.22^**^	−0.20^*^	−0.17^*^	−0.19^*^	0.20^*^	0.41^**^	–	
9. UOS_others probability of neg. events_	0.06	−0.03	0.09	−0.01	0.17^*^	0.22^**^	0.66^**^	0.30^**^	–

* p < 0.05;

** p < 0.01. UOS_own probability of pos. events_, estimate of own probability of experiencing positive events in the future; UOS_own probability of neg. events_, estimate of own probability of experiencing negative events in the future; UOS_others probability of pos. events_, estimate of others probability of experiencing positive events in the future; UOS_others probability of neg. events_, estimate of others probability of experiencing negative events in the future.

## Discussion

This study was conducted to explore and compare prospective cognitions (i.e., hope and probability estimates of future events) of never depressed (ND), previously depressed (PD), and clinically depressed (CD) individuals.

Correlational analyses for the total sample showed significant relationships between dysphoric symptoms, dysfunctional attitudes, hope, and probability estimates of future events, indicating substantial connections between these concepts. When controlled for depression severity, however, dysfunctional attitudes were solely associated with low levels of hope, but not probability estimates of future events. Hope was correlated with higher probability estimates of experiencing positive events in the future.

A multiple regression analysis showed that approximately 55% of the variance in depressive symptoms as measured by the BDI is explained by dysfunctional attitudes, hope, and probability estimates of future events. This finding underscores the importance of cognitions for the understanding of depression, dysfunctional attitudes and agency in particular.

Comparing the three groups, the results with respect to the NDs and the CDs mainly replicate previous findings reviewed above. Significant negative associations between Snyder et al.'s (1991) conceptualization of hope and depressive symptoms have been found (cf. Chang, 2003). In terms of the agency and pathways components of hope, the cognitive willpower or energy to get moving toward one's goal (hope agency) and perceived ability to generate routes to get somewhere (hope pathways) were significantly reduced in CDs compared to NDs. Furthermore, both the NDs and the CDs expected that more positive than negative events would happen to them. However, there were differences between the NDs and the CDs in the expectation of positive and negative events. In line with previous findings (e.g., MacLeod et al., 1997a), the CDs estimated the probability of experiencing positive events in the future as lower as the NDs, and they judged the probability of experiencing negative events as higher as the NDs. They also expected that positive events are more likely to happen to others than the NDs did.

The main purpose of the present study was, however, to add to the understanding of hope and optimism in previously depressed individuals. With regard to hope, as measured with the Hope Scale, the PDs fell just between the NDs and the CDs on the two components of hope. They showed higher scores on hope agency and hope pathways than the CD group, but lower scores than the ND group. With respect to expectations for the future they differed from the CDs, but not the NDs. In other words, they estimated their own and others probability of experiencing positive and negative events in the future in the same way as the NDs. Accordingly, PDs were able to form positive prospective cognitions to the same degree, as did the NDs.

These findings contribute to a better understanding of prospective cognitions in previously depressed individuals, but also have clinical implications. The results suggest that recovery from depression is not necessarily accompanied by the restoration of normal levels of hope. Both the agency and pathways components of hope were reduced in previously depressed compared to normal controls, making them possibly vulnerable to new depressive episodes. As a consequence, treatments for depression should include a focus on the patients' goal-directed determination (the agency component of hope) and their ability to find ways to meet their goals (the pathways component of hope), for example by means of interventions as outlined by Snyder (1995) or cognitive-behavioral techniques (Beck et al., 1979). Further, treatments for depression should also be evaluated in terms of their impact on hope.

Some limitations of this study should be noted. The cross-sectional design of the study prevents causal inferences about the observed relationships. Future studies may use longitudinal designs to investigate the temporal relationships between depression, hope, expectations, and the initiation and execution of actions. In addition, with the methodology used in the present study, the accuracy of the participants' estimates could not be assessed (cf. MacLeod, 1999; Harris and Hahn, 2011). However, findings from other studies (e.g., Strunk et al., 2006) suggest that the expectations of depressed individuals are not only pessimistic, but also unrealistic. Finally, as only mood disorders and psychotic symptoms were assessed in the participants, the effects of other psychological disorders often comorbid with depression (e.g., anxiety or obsessive-compulsive disorder) could not be examined.

In summary, the results of the present study give support to existing knowledge about hope and probability estimates of future events in clinically depressed and non-depressed individuals with clinically depressed being more hopeless and having less positive and more negative expectations for the future. The current investigation contributes with new findings about previously depressed individuals. Previously depressed showed reduced hope, but differed not from never depressed in their estimates for the probability of future events.

### Conflict of interest statement

The authors declare that the research was conducted in the absence of any commercial or financial relationships that could be construed as a potential conflict of interest.
